# Biological Assessment of a Calcium Silicate Incorporated Hydroxyapatite-Gelatin Nanocomposite: A Comparison to Decellularized Bone Matrix

**DOI:** 10.1155/2014/837524

**Published:** 2014-06-26

**Authors:** Dong Joon Lee, Ricardo Padilla, He Zhang, Wei-Shou Hu, Ching-Chang Ko

**Affiliations:** ^1^NC Oral Health Institute, School of Dentistry, University of North Carolina, CB 7454, Chapel Hill, NC 27599, USA; ^2^Department of Diagnostic Sciences, School of Dentistry, University of North Carolina, CB 7454, Chapel Hill, NC 27599, USA; ^3^Department of Chemical Engineering and Materials Science, University of Minnesota, Minneapolis, MN 55455, USA; ^4^Department of Orthodontics, School of Dentistry, University of North Carolina, 275 Brauer Hall, CB 7454, Chapel Hill, NC 27599, USA

## Abstract

Our laboratory utilized biomimicry to develop a synthetic bone scaffold based on hydroxyapatite-gelatin-calcium silicate (HGCS). Here, we evaluated the potential of HGCS scaffold in bone formation *in vivo* using the rat calvarial critical-sized defect (CSD). Twelve Sprague-Dawley rats were randomized to four groups: control (defect only), decellularized bone matrix (DECBM), and HGCS with and without multipotent adult progenitor cells (MAPCs). DECBM was prepared by removing all the cells using SDS and NH_4_OH. After 12 weeks, the CSD specimens were harvested to evaluate radiographical, histological, and histomorphometrical outcomes. The* in vitro* osteogenic effects of the materials were studied by focal adhesion, MTS, and alizarin red. Micro-CT analysis indicated that the DECBM and the HGCS scaffold groups developed greater radiopaque areas than the other groups. Bone regeneration, assessed using histological analysis and fluorochrome labeling, was the highest in the HGCS scaffold seeded with MAPCs. The DECBM group showed limited osteoinductivity, causing a gap between the implant and host tissue. The group grafted with HGCS+MAPCs resulting in twice as much new bone formation seems to indicate a role for effective bone regeneration. In conclusion, the novel HGCS scaffold could improve bone regeneration and is a promising carrier for stem cell-mediated bone regeneration.

## 1. Introduction

A critical-sized defect (CSD) is known as the smallest size tissue defect that will not completely heal by itself over the duration of the experiment or the life of the subject [1, 2]. A CSD caused by neoplastic, inflammatory, congenital, or traumatic etiologies requires a mechanically stiff biomaterial (scaffold) and a cell source (osteoblasts) for the effective regeneration [3–5]. Current available options to repair CSD are autografts allografts and alloplastic grafts (synthetic bone substitutes). The autograft is the gold standard, but there are only a few bones that can be used as donor tissue and frequently result in donor site morbidity. Allografts have a high risk of tissue rejection and potential viral or bacterial transmission; moreover, poor tissue integration with host tissue causes the grafting failure rate of 15 to 25% [6–8]. Since both options are highly limited by the age of the donors/recipients and their availability, the demand for synthetic bone substitutes has been increasing as an alternative source for bone regeneration.

Although hydroxyapatite (HA) is the most widely studied stiff scaffold material, the frequency of its clinical use is less than 10% of all bone grafting procedures due to its unstable fixation and insufficient interaction with host tissues [7]. Instead, hydroxyapatite composites (e.g., hydroxyapatite plus collagen derivatives) have been developed to mimic biochemical and biomechanical properties of natural bone in order to enhance osteointegration and graft healing for potential biomedical applications [9, 10]. The rapidly evolving technology enables the development of biomimetic nanocomposite biomaterials that fulfill the current requirements of an improved bone scaffold.

Recently, a new biomimetic nanocomposite, hydroxyapatite gelatin-calcium silicate (HGCS), has been developed to address the processability of the hydroxyapatite nanocomposite [11–13]. HGCS amalgamates the hydroxyapatite-gelatin (HG: degraded collagen) composite particles by* in situ* pozzolanic formation of calcium silicate (CS), which interacts among gelatin, silica, and calcium hydroxide (Ca(OH)_2_). We find that HGCS improves cytocompatibility, increases* in vitro* osteogenesis, and has a greater mechanical strength when compared to previous hydroxyapatite-collagen and hydroxyapatite-gelatin nanocomposites. Above all, the compressive strength of HGCS is significantly higher than that of HG. Notably, the CS formed by the chemical reaction between silica and calcium hydroxide affects the mechanical properties of HGCS scaffolds, which increased from 43.7 to 93.6 MPa (unpublished in-house data). Therefore, the addition of CS could enhance the mechanical strength. The performance of HGCS for* in vitro* and* in vivo* ostegenesis is, however, unknown, which fuels an additional investigation.

Another unique scaffold material is decellularized natural bone matrix (DECBM), which has received attention due to its natural origin and because it is expected to serve as a rigid scaffolding material similar to HGCS. Hashimoto et al. were the first to report a decellularization method for rat femurs using high pressure and measured the potential of cells to repopulate the bone [14]. Their evaluation was restricted to subcutaneous implantation, which requires further investigation with the* in vivo* bony defect model. The present study will develop a simplified laboratory protocol for bone decellularization and compare the applicability of decellularized calvarial bone matrix to HGCS using the rat calvaria CSD model.

Both HGCS and DECBM may be stiff enough to be utilized as scaffolds to restore bone defects. For a CSD, however, regeneration would require production of extracellular matrices by living cells. Stem cells have been known as an ideal cell source because they are readily available, nonimmunodeficient, and disease free when transplanted into patients. Bone regeneration with a cell-seeded scaffold was proven to have better regeneration potential than without cells. Bone marrow-derived mesenchymal stem cells (BM-MSCs) have been widely used and proved to have high osteogenic potential both* in vitro* and* in vivo* [15, 16]. Multipotent adult progenitor cells (MAPCs) are adult stem cells originated from bone marrow as well. MAPCs can form all three germ layers, in which mesoderm can give rise to bone tissue when dexamethasone is present [17].

Since MAPCs express higher levels of stem cell makers such as OCT-3/4, show a high proliferative rate, and can exert immunosuppressive effects on T-cell alloreactivity and proliferation, they are expected to become an alternative cell source for bone tissue engineering [18–21]. MAPCs were initially isolated from mice and rats, and specifically applied in bone tissue engineering by Ferreira et al. MAPCs have yielded positively* in vitro* osteogenic differentiation and* in vivo* bone formation [22, 23].

In the present study, we developed the HGCS scaffold and investigated its osteogenic efficiency in healing of CSDs by comparing it with DECBM (natural matrix) and CSDs with no graft as control. We also evaluated whether the bone formation process using the HGCS is related to the presence of MAPCs. Given the known positive effects of CS added to the HG scaffold for bone regeneration, the cooperative interaction between MAPCs and HGCS scaffold showed facilitated bone regeneration.

## 2. Materials and Methods

### 2.1. Preparation of HGCS Scaffold and DECBM

The HGCS scaffolds were prepared by* in situ* hybridization of CS with HG powders which were biomimetically synthesized by the coprecipitation method. The method to synthesize HAp-Gel slurry was well described in previous studies [11]. Briefly, the calcium hydroxide powder was mixed with HG powder and cross-linked with enTMOS (bis [3-(trimethoxysilyl)-propyl] ethylenediamine) for 30 seconds. To initiate the sol-gel reaction, calcium chloride solution (48 *μ*L) was added to the mixture. When the mixture began thickening, it was quickly transferred into 1cc syringes with 1 mm inner diameter needles. The HGCS paste was extruded from the syringe as intertwined threads to generate the macroporosity. The samples were dried in air for one week and then sterilized with ethylene oxide gas. To prepare the DECBM, the rat calvaria were harvested (natural bone matrix: NBM) using a trephine burr (8 mm in diameter) and washed with distilled water for 1 hour to remove blood elements. They were placed into a decellularizing solution containing 0.5% sodium dodecyl sulfate (SDS; Sigma-Aldrich, St. Louis, MO, USA) and 0.1% ammonium hydroxide (NH_4_OH: Sigma-Aldrich, St. Louis, MO, USA) and placed on a mechanical shaker at room temperature. The detergent solution was replaced every 36 hours for 3 weeks. The completion of the decellularization process was confirmed by histology (hematoxylin and eosin (H&E) staining) and DNA assay. Then, the DECBM samples were repeatedly washed with distilled water until detergents were completely removed from the matrix. The DECBM samples were sterilized using the same dose of gamma irradiation (10,000 Gy) as vascular decellularized grafts before implantation [24].

### 2.2. Mechanical Property of GEMOSIL-CS and DECBM

The compressive strength of HGCS (*n* = 10) was determined with the use of an Instron (model 4204, Canton, MA, USA) and compared to HG specimens (*n* = 10). All samples were prepared in a cylindrical shape with a 1 : 2 ratio of diameter (3.5 mm) to length (7.0 mm). The uniaxial force (0.5 mm/min) was applied to the samples for compressive strength, and five samples were evaluated per group. The result was determined from the maximum strength value on the stress-strain curve. Due to the inherent shape of the calvaria, decellularized calvaria samples were tested using the three-point bending method. The strip sample, 6.3 mm in length (L) × 6 mm in width (d) × 0.8 mm in thickness (t), was prepared and tested at the cross-head speed of 0.1 mm/min. The force was recorded in real time and peak force (F) at failure was measured. The bending flexure strength of decellularized calvaria (*n* = 10) was calculated by the formula 3FL/2*dt* ^2^. The fresh calvaria samples (NBM, *n* = 10) were tested for comparison.

### 2.3. Thermogravimetric Analysis (TGA) of DECBM

The decellularized and nondecellularized (natural) calvarial bone samples were air-dried and ground to powders. Natural calvarial bone powders were used as controls. From each group, 10 mg of powder was placed on a platinum sample holder, which was then loaded inside the Q-500 thermogravimetric analyzer (TA Instrument, New Castle, DE, USA). The test was performed in the temperature range from 30°C to 800°C with a heating rate of 5°C/min. Weight changes were recorded in terms of temperature.

### 2.4. *In Vitro* Osteogenic Study of HGCS and DECBM

MC3T3-E1 preosteoblasts were isolated from mouse calvaria and subcultured to test* in vitro* cytocompatibility and osteogenic potential for the materials coated on the 35 mm culture dishes using P-6,000 Spin Coater (Specialty Coating Systems, Inc., Indianapolis, IN, USA). Briefly, finely grounded DECBM was mixed into 1 mL of methanol and 44.4 *μ*L of enTMOS by voltexing for 15 seconds. For HGCS, HG slurry in 1 mL methanol was mixed with 29.6 mg of Ca(OH)_2_ powder and 44.4 *μ*L of enTMOS by voltexing for 15 seconds. Then 50 *μ*L from each mixture was sprayed in the center of a spinning 35 mm dish on the spin coater and spinned for 20 seconds at 6,000 rpm. The coated dishes were sterilized under UV for 24 hours and washed with PBS before being used.

#### 2.4.1. Focal Adhesion Assay

MC3T3-E1 cell cytoskeletal structures were assessed to reveal cell morphology after 3 days of cell culture on the coated dishes. Following fixation in 4% formaldehyde, the cells were permeabilized in a 0.5% Triton X-100 (Sigma-Aldrich, St. Louis, MO, USA) in PBS for 5 min. After blocking with 5% of BSA solution, the cells were incubated with rhodamine phalloidin (1 : 100 ratio in 5% BSA/PBS, monoclonal, Molecular Probes, OR, USA) and anti-*β*-tubulin primary antibody (1 : 100 ratio in 5% BSA/PBS, monoclonal anti-human, Sigma, UK) overnight at 4°C. Then, the cells were thoroughly washed in 0.05% Tween 20 in PBS and incubated with FITC conjugated secondary antibody (1 : 50 ratio in 5% BSA/PBS, Vector Laboratories, UK) for 1 h at 4°C. The nuclei of cells were stained with VECTASHIELD^®^ mounting medium with DAPI (Vector laboratories, Inc., Burlingame, CA USA) and images were acquired using a fluorescence microscope (Nikon Eclipse Ti-U, Nikon Instruments, Melville, NY).

#### 2.4.2. MTS Assay

The proliferation of the MC3T3-E1 cells on the coated dishes was conducted using MTS assay as instructed in company manual. The MTS (3-(4,5-dimethylthiazol-2-yl)-5-(3 carboxymethoxyphenyl)-2-(4-Sulfophenyl)-2H-tetrazolium) (Promega Co., Madison, WI, USA) reacted with cells for an hour. Then absorbance of each group was measured on days 1, 3, 5, 7, and 9, respectively, at 490 nm using a Plate reader (Biorad, Hercules, CA, USA).

#### 2.4.3. Mineralization Assay

For differentiation, MC3T3-E1 cells were cultured directly on the HGCS- and DECBM-coated 35 mm dishes with osteogenic media for 4, 7, and 21 days. After washing with PBS, the cells were fixed for 10 minutes in formalin, washed, stained with 40 mM Alizarin Red (Acros Organics, Geel, Belgium) at pH 4.2 for 10 minutes, rinsed with deionized water 6 times, and air-dried. Mineralized nodule images were obtained with a Nikon Eclipse Ti-U microscope (Nikon Instruments Inc., Melville, NY, USA).

### 2.5. *In Vivo* Implantation of Scaffolds

Rat MAPCs were isolated, expanded, and seeded onto the HGCS scaffolds (*n* = 3). The isolation, culture, and seeding on the scaffold of rat MAPCs were described in our previous study [23]. Twelve male Sprague-Dawley rats (Charles River, Wilmington, MA, about 250 g, 7 weeks) were initially anesthetized by intramuscular injection of 10 mg/kg Ketamine-HCl (Puteney Inc., Portland, ME, USA) and additional Ketamine was administered during experiment as needed. After shaving and sterilizing the surgical site, an approximately 2 cm mid-sagittal skin incision was made from occipital to frontal scalp, and the subcutaneous tissue was dissected and reflected to each side with the periosteum to expose the osseous surface of the skull. An 8 mm CSD was created without damaging the underlying dura and mid-sagittal blood vessels using a low-speed dental trephine burr. Four treatment groups with three rats each underwent surgery for a total of 12 CSDs. The small sample size (*n* = 3) was chosen because this was a screening/pilot test for a series of developing biomaterials as indicated in our previous reports [23]. Three experimental groups used different types of scaffolds (DECBM, HGCS seeded with MAPCs, and HGCS). The control group consisted of CSDs with no grafting at all. After implantation of the scaffold materials, the periosteum was closed with 4–0 chromic gut suture, and then skin was closed with 3–0 silk suture. To prevent postsurgical infection, 20 mg/kg of cefazolin (Hospira Inc., Lake Forest, IL, USA) was injected intramuscularly once per day for 7 days after surgery. For the mineral apposition rate (MAR) measurement, fluorochrome labels such as Alizarin Red-S (30 mg/kg, Sigma Aldrich, St. Louis, MO, USA) and Calcein (20 mg/kg, Sigma Aldrich, St. Louis, MO, USA) were injected perivascularly to each animal twice during the study. Alizarin Red was administered 10 days after the surgery and calcein was given 15 days before sacrifice. The interlabeling periods were 10 and 70 days for rats sacrificed at 12 weeks.

### 2.6. Micro-CT Analysis

Rats were sacrificed 12 weeks after scaffold implantation. The calvaria were excised carefully by preserving the implanted sites and then fixed in 10% formaldehyde for 7 days at 4°C. Subsequently, they were transferred into 70% isopropyl alcohol. The calvaria explants were scanned by using a micro-CT system (mCT 40; Scanco Medical, Brüttisellen, Switzerland) at 70 kV and 114 mA with a 200 ms integration time. Detailed setting parameters for acquisition and analysis of the acquired images were described in a previous study [15]. After 3D reconstruction, the percentage of radiopaque areas in the defects was measured using the Nikon NIS Elements software and tabulated by treatment group. The radiopacity (%) was obtained by dividing the new radiopaque areas in the CSD by the total defect area. The data were presented as average ± standard deviation. One-way ANOVA was used to compare the means among four groups.

### 2.7. Mineral Apposition Rate (MAR) by Fluorescence Microscopy

Harvested calvaria specimens were dehydrated and subsequently infiltrated with resin (Technovit, Heraeus Kulzer GmbH, Germany) for 23 days. The method for the slide preparation was described previously [20]. The polished sections were viewed using a Nikon fluorescence microscope apparatus with bright field, TRITC and FITC filters, and a Nikon Eclipse Ti-U digital camera (Nikon Instruments Inc., Melville, NY, USA). Each fluorescent image was merged to measure the distance between fluorescent labeled layers at the interface between native bone and the implanted scaffold (INBS) and at the central pore area with the Nikon NIS Elements software (Nikon Instruments Inc., Melville, NY, USA) tools. A total of five measurements were made along the span of each double label. The MAR was calculated using the following equation: MAR (*μ*m/day) = ∑_*χ*_/*nt*, where ∑_*χ*_ is the sum of all the measurements between double labels, *n* is the total number of measurements, and *t* is the time (days) interval expressed [25–27]. The mean distance of the five regions was subject to statistical analysis and the overall mean distance was calculated for each group. Two-way ANOVA was used to compare the means among four groups.

### 2.8. Histological Determination for New Bone Tissue Formation

After analysis for MAR study, calvaria specimen slides were further stained with Steven's Blue by counterstaining with Van Gieson to visualize the formation of newly formed bone (NFB) tissue for the quantitation as previously described [23]. Briefly, entire images of the medial (central) sagittal histologic section were acquired with a DP70 color digital camera equipped with color image software (DP11, Olympus USA, Center Valley, PA, USA) under 20x magnification and then merged using Adobe Photoshop CS6 (Adobe Systems Inc., San Jose, CA USA) to recreate as one figure. The new bone surface area (B.Ar.) and the total area of each defect (T.Ar.) were measured in pixels by using an automated image analysis system (Image J software version 1.46R, NIH, Bethesda, MD, USA) to calculate the NFB (in %: B.Ar./T.Ar/0.01) based on the standardized protocols of the American Society for Bone and Mineral Research [28]. The one tail Student* t*-test was used to compare the means between the groups.

## 3. Results and Discussion

The two main components of tissue engineering constructs for* in vivo* implantation are the cells and the scaffold material. Since the bone regenerating potential of MAPCs was well reported in previous studies [18, 23], our efforts were focused mainly on the materials aspect to compare natural and synthetic components in bone regeneration. Previously, HG was developed by mimicking natural bone matrix (hydroxyapatite and collagen derivative) with promising properties for bone regeneration [29]. However, the weak bonding force between HG particles and siloxane was prone to brittle fracture and resulted in a relatively low compressive strength when compared to natural bone. Applying CS to HG created a new scaffold material, HGCS, which not only reinforced its mechanical strength but also improved osteoblast adhesion, proliferation, and differentiation by the stimulation of released calcium ions. Another value of this study was to apply a naturally derived biomaterial via removing cellular components from the matrix for the bone regeneration. DECBM was expected to be an ideal candidate for a scaffold due to its function as supporting structure and regulator of cellular functions such as cell viability, proliferation, and differentiation [30–33]. Indeed, the natural ECM is known to have a modulatory effect of signal transduction triggered by bioactive molecules (growth factors and cytokines) [34, 35]. It could be preserved even after decellularization, as described in previous applications on various tissues [36, 37]. Here, we developed an allogenic natural bone scaffold using a decellularization technique to compare it with a synthetic scaffold (HGCS) for the potential of* in vivo* bone regeneration.

### 3.1. Characterization of DECBM and HGCS

**Figure 1:**
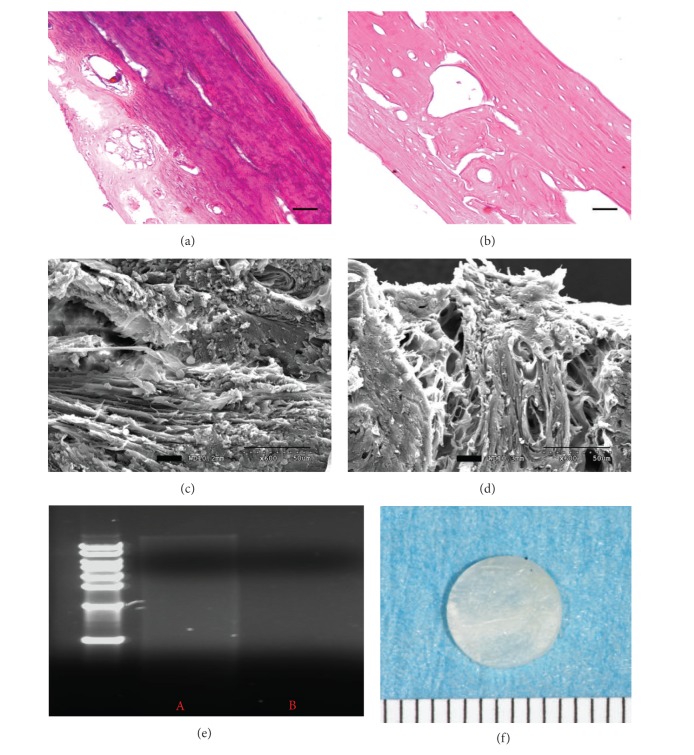
Decellularized rat calvaria. Hematoxylin and eosin-stained cross-section of calvaria before (a) and after (b) decellularization (scale bar: 50 *μ*m). SEM analyses visualize the presence of cells in NBM (c) and the maintained ECM structure after decellularization (d); scale bar: 50 *μ*m. DNA assay was used to detect any residual DNA before (lane A) and after the decellularization of calvaria (lane B). Gross image of rat calvaria after the decellularization process (f); scale: 1 mm.

The duration of the decellularization process for rat calvaria was optimized by 0.5% SDS and 0.1% NH_4_OH dissolved in distilled water [38, 39]. This process effectively disrupted cell membranes and cleaved DNA to minimize immunological rejection. The completion of decellularization was confirmed by histology and DNA assay. After 2 weeks, H&E staining of the decellularized tissue revealed absence of both nuclei and cytoplasmic compartments (Figures 1(a) and 1(b)) and no DNA was detected from agarose gel electrophoresis (Figure 1(e)). Removing the cells from the matrix is an important step because it may prevent humoral immune reactions against membrane proteins. It is also important to completely remove DNA from the matrix because it can stimulate the immune system by activating cytokine production and B-cell immunoglobulin secretion after allogenic or xenogenic implantation [40]. Scanning electron microscope (SEM) evaluation revealed that both matrices exhibited similar morphology in the distribution of collagen and mineralized fibers residing in the matrix (Figures 1(c) and 1(d)). In NBM cells are attached and embedded into the matrix; in DECBM cells are absent, confirming the effective decellularization from the calvarial bone tissue.

**Figure 2:**
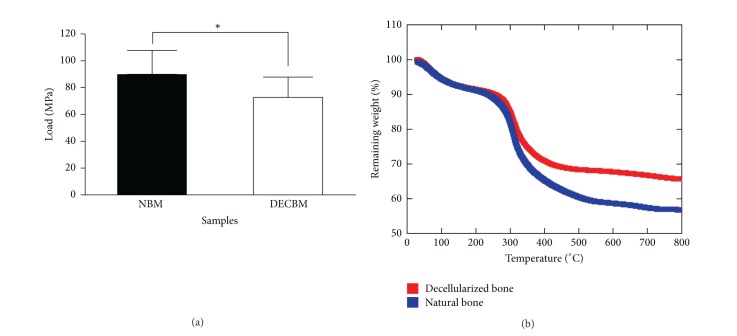
Three-point bending test of natural bone matrix (NBM) versus decellularized bone matrix (DECBM) of calvaria with a sample size *n* = 5 (a). Samples were maintained hydrated with PBS during experiment. The strength was decreased by 19.42 MPa after decellularization (*P* < 0.05). A typical TGA result showed the weight loss versus temperature. Distinct weight drop patterns are attributed to the degradation of water (initial drop in the curve prior to 100°C) and organic materials (second drop at 300) (b).

The TGA data reveals the typical pattern of mineral tissues with a higher inorganic to organic ratio in DECBM than in NBM (Figure 2(b)). Absorbed water inside the DECBM and NBM could be removed completely by heating to 200°C. The weight loss of the samples was attributed to the thermal degradation of proteins including collagen, noncollagenous proteins, and cell membranes (300–560°C) [41]. The NBM samples indicated a continuous weight drop from 300 to 550°C. The DECBM samples showed an abrupt weight drop from 300°C to 400°C, ceased at 450°C, and then followed with a gradual weight drop until 600°C. The final weight remainder represents the percentage of inorganic content in the matrix. In fact, the final weight % of DECBM (65%) was greater than that of NBM (58%), indirectly verifying that approximately 7% of the organic contents were removed through the decellularizing process.

The integrity of the bone matrix directly associated with mechanical strength was measured by the three-point bending test and the compression test in DECBM and HGCS, respectively. The mean values of the three-point bending test were 72.00 ± 14.14 MPa and 89.36 ± 17.34 MPa for decellularized and nondecellularized bones, respectively (Figure 2(a)). The decellularization process decreased the mechanical strength by 19.42% (*n* = 10, *P* < 0.05) in DECBM compared to NBM. The difference was possibly caused by an enzymatic effect on the structural proteins and more likely due to a loss of soluble protein by SDS during the decellularizing process. SDS was considered to be a better choice than nonionic agents to thoroughly remove soluble proteins from the matrix in such a hard and compact tissue like bone (in-house nonpublished data). The compressive strength of HGCS was significantly higher than that of HG. Certainly, CS played a role in increasing this mechanical property of the HGCS scaffold from 43.7 to 93.6 MPa (in-house nonpublished data). This reinforcement is attributed to the* in situ* formation of CS due to the chemical reaction between silica and calcium hydroxide. Therefore, both DECBM and HGCS implants can provide strength approaching that of natural bone though the values were still inferior to natural bone.

### 3.2. *In Vitro* Osteoblasts Attachment, Growth, and Osteogenic Differentiation on DECBM- and HGCS-Coated Dishes

**Figure 3:**
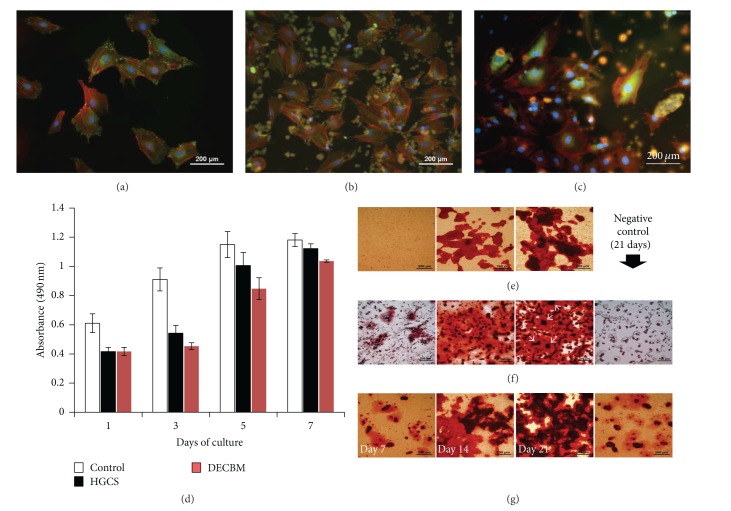
*In vitro* assessment of osteoblast activity on the material-coated dishes. (1) Focal adhesion assay of MC3T3 cells on the coated culture plate with no coating as control (a), HGCS (b), and DECBM (c) after 3 days of culture. MTS assay for MC3T3-E1 cell proliferation on the coated culture plate with DECBM, GEMOSIL-CS, and no coating as control on 1, 3, 5, and 7 days of culture (d). Mineralized nodules were detected by Alizarin Red staining after culturing MC3T3 cells with osteogenic media on the coated culture plate with no coating as control (e), HGCS (f), and DECBM (g) after 7, 14, and 21 days. Negative control was also tested as coated culture plate without cells for 21 days with osteogenic media.

To understand osteoblast-scaffold interactions, their* in vivo* bioactivity, and cell integration within the scaffold material during bone regeneration, the materials were assessed* in vitro* by culturing osteoblasts directly on HGCS and grounded DECBM-coated dishes. Prior to use, the quality of the coating was confirmed by observing them under a microscope for even distribution of the materials and consistency of coating. In the control group (no-coating), the focal adhesion assay with MC3T3-E1 preosteoblasts generally indicated a round cell morphology in which their actin filaments were spread out in multiple directions by attaching with well-distributed vinculins in the dish (Figure 3(a)). Cells grown on the DECBM- and HGCS-coated dishes demonstrated less spreading but still had similar morphology as the control group cells. Most of the vinculins were observed near or on the material particles, which supports DECBM and HGCS as being good substrates for cell attachment (Figures 3(b) and 3(c)). MTS assay and mineralization studies were performed for* in vitro* osteogenic potential with MC3T3-E1 cells on material-coated dishes. Since a higher number of cells represent a higher formazan activity, we analyzed the MTS assay for the cell proliferative potential by measuring the formazan activity over time. Figure 3(d) shows the MTS activity of the MC3T3-E1 cells measured on days 1, 3, 5, and 7. The number of cells on both DECBM- and HGCS-coated dishes increased up to 7 days. Cells on HGCS-coated dishes showed a higher proliferative potential than those on DECBM, but less potential than those of the control group between days 1 and 7. The lower growth potential on DECBM compared to the control group was partially due to the growth behavior of the cells on the coated matrix. The topography of DECBM coatings would delay the cell growth in early time points due to an increased surface area, but the cells grew similarly to the control group after 7 days. The cells on DECBM grew by aggregating around DECBM particles and formed thread-like collagen structures (data not shown), which resulted in a multilayered cell culture, unlike HGCS and control groups.

Mineralization was evaluated by nodule formation on days 7, 14, and 21 after differentiation with the osteogenic media (*α*-MEM with 10 mM *β*-glycerophosphate (Sigma-Aldrich, St. Louis, MO, USA) and 0.2 mM ascorbic acid (Sigma-Aldrich, St. Louis, MO, USA)). In the controls, calcium nodules were visible 14 days after differentiation and became darker and larger at 21 days, consistent with the typical mineralization pattern of MC3T3-E1 cells under our differentiation conditions (Figure 3(e)). DECBM-coated dishes showed earlier nodule formation and overall were more intense and larger than in the control dish at days 14 and 21 (Figure 3(g)). However, the microscopic pattern of calcium deposition on the HGCS-coated dish was very different than on the DECBM and the control dishes (Figure 3(f)). Cells on the HG started to form small nodule particles, which then enlarged and surrounded the Ca(OH)_2_ particles (white arrows). Alizarin Red staining revealed that Ca(OH)_2_ particles were located in the middle of most nodules and likely promoted osteogenic differentiation by providing calcium ions. Ca(OH)_2_-coated dishes without cells were also stained to serve as negative control.

### 3.3. *In Vivo* Bone Regeneration (Mineral Apposition Rate)

**Figure 4:**
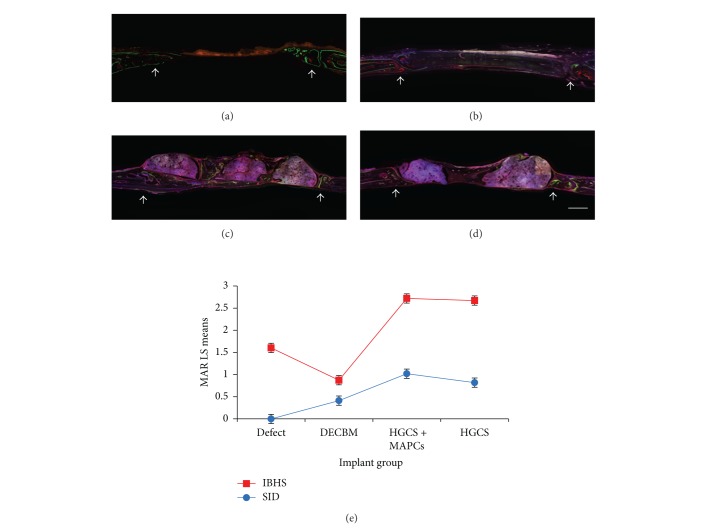
Fluorescence image of histological cross-section represented fluorochrome-labeled mineral deposition in defect (a), DECBM (b), HGCS with MAPCs (c), and HGCS (d). Calcein (green) and Alizarin Red (red) were administered in series. Mineral apposition rate (MAR) at the interface between host bone-scaffold (IBHS) and inside scaffold in the defect (SID) was determined at 12 weeks after implantation of scaffolds (e). Data are shown as mean ± SD of three independent experiments, *P* < 0.05 (scale bars: 1 mm). White arrows represent the margin of the defect.

Four groups were tested for bone regeneration in the rat calvaria critical-sized defect model: empty defects (control group), DECBM, HGCS scaffolds seeded with MAPCs (HGCS+MAPCs), and HGCS scaffolds only. After 12 weeks of postimplantation, the entire calvarial was harvested for MAR, gross, radiographic, and histomorphometric analyses. To evaluate the bone formation rate, MAR was assessed by measuring a total of six randomly selected points along the span of each scaffold. These were double-labeled at both the interface between the host tissue and the scaffold (IBHS) and in the middle of the defect where healing bone infiltrated into the porous scaffold (SID). The results of fluorochrome labeled histology (a to d) and the MAR measurements (e) are presented in Figure 4. At the IBHS, both HGCS+MAPCs and HGCS groups demonstrated higher MAR values (2.72 ± 0.34 *μ*m/day and 2.67 ± 0.2 *μ*m/day, resp.) than the empty defect control group, which had MAR of 1.6 ± 0.53 *μ*m/day. In addition, the MAR value in the DECBM group was 0.87 ± 0.07 *μ*m/day, which was the lowest rate of mineral apposition. At the IBHS of the DECBM group, only a few areas expressed the fluorochromes, signifying that osteoblasts were scantly present and that there was low osteoblastic activity. According to the previous study by Parfitt et al., the total rate of bone formation was affected by the number of osteoblasts and the average volume of matrix secreted by the osteoblasts [42, 43].

The highest MAR in the SID was in the HGCS+MAPCs group (1.02 ± 0.23 *μ*m/day). The MAR in the HGCS-only group (0.82 ± 0.1 *μ*m/day) was greater than in the DECBM group (0.41 ± 0.1 *μ*m/day). The difference in MAR between the HGCS group and the HGCS+MAPCs group may be due to the MAPCs differentiating into osteoblasts* in vivo*, therefore increasing their activity and the rate of bone mineral apposition in the SID. The MAR of the DECBM group in the SID can only be measured on the surface of the DECBM and not inside of the scaffold. In the control group, the MAR in the SID could not be calculated because the empty defects did not heal after 12 weeks (Figure 4(e)).

Overall, the HGCS+MAPCs and HGCS groups showed increased MAR over the DECBM and the empty defect (control) groups. In addition, the MAR in the DECBM group was only detectable on the surface of the DECBM. Fluorochromes were observed to be deposited only on the surface of the DECBM scaffold because DECBM has inadequate pores; there was no cell migration into the matrix to regenerate bone. In the HGCS with and without MAPCs groups, fluorochrome labels could be seen extensively with measureable distances, whereas the empty defect group had them only at the periphery of the defect. Although MAR was a significant indicator for mineral apposition rate at a specified timeframe, further assessment by histomorphometry and radiographic analysis can provide more information regarding the total new bone formation (NBF).

### 3.4. *In Vivo* Bone Regeneration (Histomorphometry and 3D Micro-CT Evidence)

**Figure 5:**
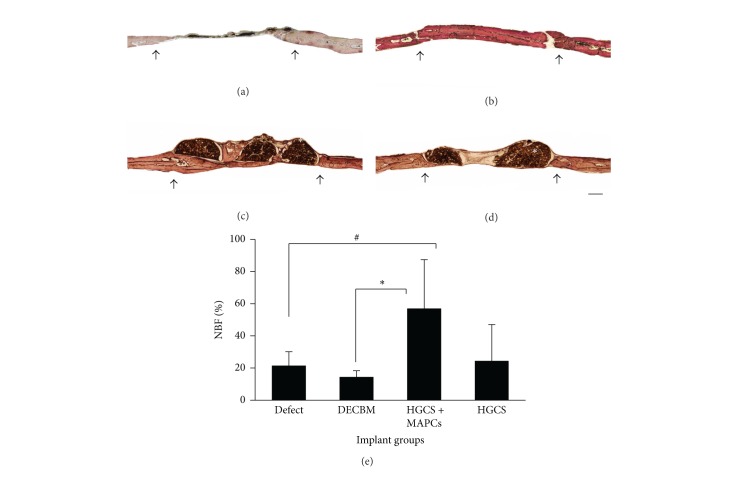
Histological sections were processed for sagittal section of the defect area after 12 weeks of implantation and stained with Steven Blue and Van Gieson. The representative section was shown from each group of defects (a), DECBM (b), HGCS with MAPCs (c), and HGCS (d). The area of new bone formation (NFB) was quantified in % using Image J software (e). White asterisk indicates HGCS material, *P* < 0.05 (scale bars: 1 mm). Black arrows represent the margin of the defect.

**Figure 6:**
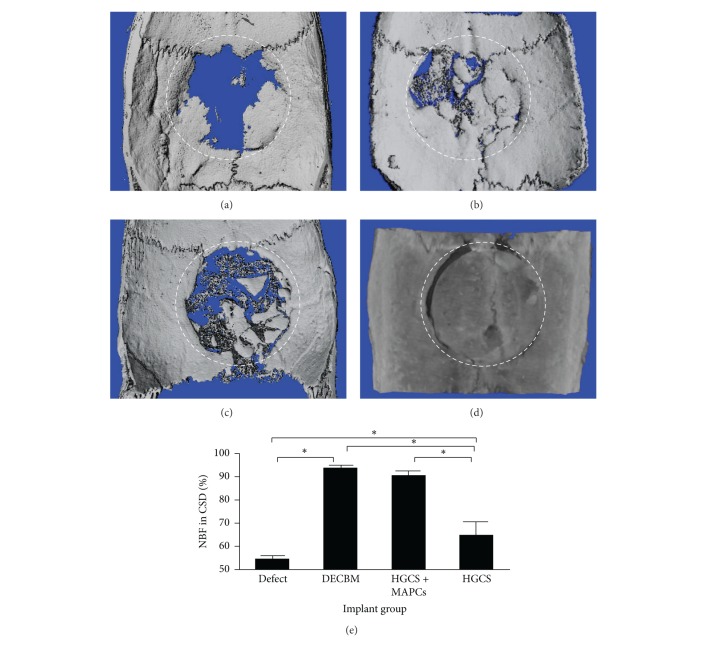
Micro-CT images of critical-sized calvarial defects at 12 weeks. Panels represent empty defect (a), DECBM (b), HGCS with MAPCs (c), and HGCS groups (d). Radiopaque area in the defect was calculated in % (e). Data were represented as mean ± STD (*n* = 3, *P* < 0.05).

After fluorochrome imaging, the undecalcified resin sections were stained with Steven Blue and Van Gieson histochemistry to identify new bone formation (fresh red) in the defect site. Histological evaluation demonstrated that the defect within the HGCS+MAPCs group was filled with new bone that bridged with the host bone by 12 weeks. In the HGCS scaffold-only group new bone was moderate, primarily at the periphery of the defect (Figure 5(c)). From all perspectives, bone regeneration was prominently better in the HGCS+MAPCs group (Figure 5(d)). At 12 weeks of postimplantation, quantitative measurements of NBF demonstrated 56.99 ± 30.44% bone regeneration for the HGCS+MAPCs group, 24.44 ± 3.94% for the HGCS-only group, 14.45 ± 3.94% for the DECBM group, and 21.51 ± 8.60% for the empty defect group. Notably, the newly formed bone in both the HGCS-only and the HGCS+MAPCs groups was well integrated at the interface between host bone and the scaffold. In the middle section of the HGCS-only group, the NBF in the area of macrospores had little calcified tissue; instead fibrous tissues were seen (Figure 5(c)). Contrastingly, the macroporous spaces in the HGCS+MAPCs were filled with newly formed bone tissue (Figure 5(d)). The importance of cells in repairing bone lesions has been documented [16, 18, 23]. In this study we confirm that NBF with HGCS scaffolds also relies on the ability of cells to increase regional bone regeneration, especially in the central area of the CSDs apart from host tissue (Figure 5(c)). The percentage of NBF in the DECBM group was significantly lower than that of the HGCS scaffold alone at 12 weeks (Figure 5(b)). Notably the empty group also promoted NBF above that of DECBM group (Figure 6(e)). Both the empty and HGCS groups relied on the host bone as a cell source. The host cells may have produced approximately 23% of NBF when there was an available space (either defect or scaffold bridging). There was an absence of NBF between DECBM and host tissue with intervening fibrous tissue. In the middle of section, the NBF was slightly formed along the outer surface of the DECBM with little osteointegration (Figures 5 and 6). This demonstrates that DECBM of calvarial bone does not allow cell infiltration.

In Figure 6, micro-CT analysis shows 3D reconstructed calvaria at 12 weeks after implantation (a to d). The percentage of radiopaque area in the defect was calculated (e). While the DECBM group showed the highest radiopaque area (94.1 ± 0.31%), the HGCS+MAPCs scaffolds revealed greater radiopaque areas (90.58 ± 0.98%) than HGCS scaffolds alone (64.76 ± 5.53%) or empty defects (54.7 ± 0.79%). The micro-CT data, except for the DECBM group, were consistent with the histological measurements providing an indirect validation of NBF. The reason for the DECBM group difference is due to the DECBM having the same inorganic matrix content as natural bone (Figure 6), making it difficult to differentiate newly formed bone from DECBM radiographically.

Taken together, our* in vivo* analyses confirmed that HGCS is stiff enough to support porous spaces for cell ingrowth and is compatible with MAPCs for osteogenic differentiation. The use of HGCS+MAPCs could facilitate new bone formation and further advance the success of bone regeneration. Although DECBM showed a higher osteogenic differentiation potential than HGCS* in vitro*, its application as 3D structural scaffold is limited due to the inability for cellular migration into the compact structure of bone.

## 4. Conclusion

Our data support that HGCS possesses osteoinductive properties as a novel synthetic biomaterial for bone regeneration and that seeding it with MAPCs yields a synergic effect to enhance bone regeneration in rat calvarial critical-sized defects. This study also confirms that the natural biomaterial, DECBM, is not suitable as a bone scaffold. Our findings suggest that further studies are needed to adapt optimal microarchitectures of HGCS to promote bone regeneration and to find alternative applications of DECBM as a scaffold in bone regeneration.
